# Analysis of the clinical characteristics and prognosis of adult *de novo* acute myeloid leukemia (none APL) with *PTPN11* mutations

**DOI:** 10.1515/med-2023-0830

**Published:** 2023-11-03

**Authors:** Li Sheng, Yajiao Liu, Yingying Zhu, Jingfen Zhou, Haiying Hua

**Affiliations:** Wuxi School of Medicine, Jiangnan University, Wuxi, Jiangsu, 214122, China; Department of Hematology, Affiliated Hospital of Jiangnan University, Wuxi, Jiangsu, 214122, China; Nursing Department, The Second Affiliated Hospital, Zhejiang University School of Medicine, Hangzhou, Zhejiang, 310000, China

**Keywords:** acute myeloid leukemia, *PTPN11* mutations, prevalence, clinical characteristics, prognosis

## Abstract

We discuss the clinical characteristics and prognostic significance of adult individuals with *PTPN11* mutations who have developed acute myeloid leukemia (AML) (none acute promyelocytic leukemia). Next generation sequencing and Sanger sequencing were used to detect 51 gene mutations, and multiplex-PCR was used to detect 41 fusion genes from 232 *de novo* adult AML patients retrospectively. About 7.76% patients harbored *PTPN11* mutations, 20 *PTPN11* alterations were identified, all of which were missense mutations in the N-SH2 (*n* = 16) and PTP (*n* = 4) domains located in exon 3. Patients with *PTPN11* ^mut^ had significantly higher platelet counts and hemoglobin levels (*p* < 0.001), which were mainly detected in M5 (*n* = 12, 66.67%, *p* < 0.001) subtype. Patients with MLL-AF6 positive showed a higher frequency of *PTPN11* ^mut^ (*p* = 0.018) in the 118 AML cases. *PTPN11* ^mut^ were accompanied by other mutations, which were *NPM1* (44.44%), *DNMT3A* (38.89%), *FLT3* (38.89%), and *NRAS* (17.2%). *PTPN11* ^mut^ had a negative impact on the complete remission rate in M5 subtype patients (*p* < 0.001). However, no statistically significant effect on overall survival (OS) with *PTPN11* ^mut^ patients in the whole cohort and age group (*p* > 0.05) was observed. Further analysis revealed no significant difference in OS among *NPM1* ^mut^/*PTPN11* ^mut^, *NPM1* ^mut^/*PTPN11* ^wt^, *DNMT3A* ^mut^/*PTPN11* ^mut^, and *DNMT3A* ^mut^/*PTPN11* ^wt^ patients (*p* > 0.05). Multivariate analysis showed the proportion of bone marrow blasts ≥65.4% was a factor significantly affecting OS in *PTPN11* ^mut^ patients (*p* = 0.043).

## 1 Introduction

Acute myeloid leukemia (AML) is a clonal malignant proliferative disease of primitive cells in the hematopoietic system, and whole-genome sequencing has revealed the complexity and high heterogeneity of AML [1]. Previous studies have indicated that approximately 86% of AML patients carry two or more gene mutations [2]. Next-generation sequencing (NGS) technology has played a pivotal role in molecular diagnostics and is increasingly being utilized in the field of hematological malignancies, particularly in the detection of gene mutations in AML patients [3,4]. NGS has led to the discovery of a growing number of AML-associated gene mutations, such as *FLT3*, *TP53*, *RUNX1*, and so on [2]. High throughput, high sensitivity, and low cost of this technology make it a valuable tool for examining the molecular pathogenesis of hematological malignancy, assisting in clinical diagnosis and therapy, and facilitating the integration of precision medicine [3,4]. AML-associated gene mutations have become crucial determinants for AML diagnosis, risk stratification, and selection of treatment strategies [5]. However, the clinical relevance of some genes in AML is still unclear and requires exploration of their clinical characteristics and prognostic significance. This will be advantageous for gaining a deeper understanding of the biological characteristic in AML, identifying potential therapeutic targets, and ultimately improving patient prognosis.

The *PTPN11* gene, identified as the first oncogene encoding a protein tyrosine phosphatase, has been found in numerous tissues and cells [6]. The *PTPN11* gene, located on chromosome 12q24, serves as a crucial regulatory factor in the RAS signaling pathway [7,8]. It comprises exons 1–16 and encodes a non-receptor protein tyrosine kinase called SHP2. SHP2 has been discovered to play a key role in the development of normal hematopoietic cells [7,8]. Research has demonstrated that SHP2 promotes the signaling cascade of the ERK pathway, while inhibiting stem cell self-renewal and differentiation in the JAK/STAT3 pathway [6]. In cytokine-dependent hematopoietic cell lineages, SHP2 has been proven to be involved in signal transduction pathways triggered by interleukin-6 (IL-6), interleukin 3/granulocyte-macrophage colony-stimulating factor (GM-CSF), leukemia inhibitory factor, and other factors [9–11]. Furthermore, SHP2 augments the robustness and fidelity of IL-6-induced JAK/STAT signaling [11].

Mutations in the *PTPN11* gene are associated with various developmental disorders, hematologic malignancies, and solid tumors, playing distinct biological roles in different mechanisms of cancer development [12,13]. Through the PI3K/AKT/GSK3β signaling pathway, *PTPN11* has been shown to promote the proliferation of breast cancer cells, making it a potential factor in carcinogenesis or progression [14]. Additionally, mutations in *PTPN11* have been found to be responsible for the development of Noonan syndrome (NS) and juvenile myelomonocytic leukemia (JMML) by triggering the RAS/MAPK signaling pathway [15,16]. The mechanism of *PTPN11* mutations in adult AML is not well understood and may be related to mutation accumulation or disruption of intracellular signaling pathways [17].

The French–American–British (FAB) classification system categorizes AML into eight subtypes based on the morphology of leukemic cells, spanning from M0 (acute myeloblastic leukemia with minimal differentiation) to M7 (acute megakaryoblastic leukemia), alongside several intermediate subtypes. Notably, the treatment regimen and prognosis differ for the M3 subtype, known as acute promyelocytic leukemia (APL). When compared to other subtypes, APL exhibits a more favorable prognosis [18,19]. According to the literature, *PTPN11* mutations are more commonly observed in the M5 subtype of pediatric AML [20,21]. The M5 subtype, also known as monocytic AML, is a specific subtype of AML characterized by the presence of abundant monocytic cells in both the bone marrow and peripheral blood [18]. *PTPN11* mutations can also occur in other subtypes of adult AML or other types of leukemia, but they are relatively less common. Papaemmanuil et al. [2] found that *PTPN11* mutations occur in less than 5% of adult AML cases, and are more frequently observed in patients with M4/M5 subtypes [22]. However, currently there is no reported evidence from domestic or international studies regarding the prognostic value of M5 subtype in patients with *PTPN11* mutations. *NPM1* is a protein widely expressed in the nucleolus, and *NPM1* mutations are considered the most common genetic alterations in AML. Due to its unique clinical features, gene expression profile, and immunophenotype, *NPM1* mutations were recognized as an independent disease subtype in the 2017 WHO classification of myeloid neoplasms and acute leukemia, with favorable prognostic significance [23,24]. Furthermore, a few studies have suggested a correlation between *PTPN11* and *NPM1* mutations, but the impact of this co-mutation on the prognosis of AML patients remains unclear [25,26]. *DNMT3A*, also known as DNA methyltransferase 3A, is a protein enzyme associated with genetic modifications. AML patients with mutations in the *DNMT3A* gene exhibit poor prognosis [27,28]. Additionally, while studies have shown that double mutations in *PTPN11* and *DNMT3A* reduce survival in mice [29], there is no evidence to support a similar impact on clinical outcomes in adult AML patients. The specific role of *PTPN11* gene mutations in adult AML remains inadequately explored. Understanding the significance of *PTPN11* mutations in adult AML can help identify novel therapeutic targets and develop more effective treatment strategies for adult AML patients.

Therefore, in this study, we conducted a retrospective analysis of gene sequencing data from 232 adult AML (none APL) patients to examine the presence of *PTPN11* mutations. We aimed to investigate the clinical characteristics of *PTPN11* gene in adult newly diagnosed AML patients from both *PTPN11* wild-type and *PTPN11* mutant perspectives. Additionally, we analyzed the co-occurrence of *PTPN11* mutations with *NPM1* and *DNMT3A* mutations, as well as the impact of M5 subtype on the prognosis of patients with *PTPN11* mutations in adult AML.

## 2 Patients and methods

### 2.1 Patients and gene sequencing

This was a retrospective analysis of gene mutations in 232 adult *de novo* AML patients (none APL) admitted to the Affiliated Hospital of Jiangnan University, Changzhou Second People’s Hospital, Wuxi People’s Hospital, and Wuxi Second People’s Hospital from January 2017 to July 2022. The study categorized the patients according to the FAB classification: M0 (*n* = 3), M1 (*n* = 23), M2 (*n* = 83), M4 (*n* = 50), M5 (*n* = 59), M6 (*n* = 3), and 11 cases were unknown. Bone marrow transplant patients were not included in the study. The sample comprised of 125 males and 107 females with a median age of 48 (18–72) years. All patients were diagnosed according to the 2016 revised World Health Organization classification criteria for hematopoietic and lymphoid tissue tumors [30,31].

To assess the gene mutations, 2 mL of bone marrow suspension was taken from each patient at the first diagnosis and extract intracellular DNA, use amplification method for library construction, bridge expansion using Illumina sequencing platform, generate a cluster, and then perform sequencing to detect AML related 51 gene mutations (the average sequencing depth was 1,000×): *PTPN11, CBL, NRAS, KRAS, RUNX1, RUNX2, CEBPA, TP53, BCOR, BCOR1, BCORL2, GATA2, SETBP2, FLT3, JAK1, JAK2, JAK3, ABL1, C-KIT, NF1, TET2, WT1, IDH1, IDH2, ASXL1, ASXL2, NPM1, CSF3R, SETD2, KMT2A, EZH2, PHF6, DNMT3A, NOTCH1, U2AF1, ETV6, CSMD1, PDGFRB, MYC, IKZF1, SETBP1, EED, ETNK1, CSF1R, FAT1, KMT2C, APC, MPL, EP300, ARID2, SRSF2, STAG2.* Data were read by selecting mutations on exons and removing both synonymous and polymorphic mutations. The first-generation PCR combined with Sanger sequencing was also utilized for supplemental testing of *FLT3-ITD*, exon 12 of the *NPM1* gene, as well as the two functional domains (TAD and BZIP) of *CEBPA*.

We analyzed the patient’s chromosomal karyotype by extracting 2–4 mL of heparin anticoagulated bone marrow suspension at initial diagnosis, and performing short-term culture for 24 h followed by conventional R-banding technique. We examined an average of 20 metaphase spreads and named the cell karyotype according to the International System for Human Cytogenetic Nomenclature (ISCN 2009). The risk classification was performed based on the chromosomal karyotype analysis results according to the European LeukemiaNet (ELN 2017) risk categories. Furthermore, RNA was extracted from the bone marrow mononuclear cells of patients using TRIZOL method in order to detect 41 common fusion genes. The reaction solution was prepared according to the instructions of the leukemia fusion gene detection kit, and the Thermo Fisher ABI7500 amplification instrument was used for the amplification reaction.

### 2.2 First induction therapy

In this study, we evaluated the efficacy of Ara-C and IDA/DNR-based chemotherapy in the treatment of AML. Patients aged less than 60 years were treated with a standard dose of Ara-C 100–200 mg/(m^2^·d) × 7d combined with idarubicin (IDA) 12 mg/(m^2^·d) × 3d or DNR 60–90 mg/(m^2^·d) × 3d. Elderly patients aged 60 and above were treated with a standard dose of Ara-C 100 mg/(m^2^·d) × 7d combined with IDA 8–12 mg/(m^2^·d) × 3d or DNR 40–60 mg/(m^2^·d) × 3d. The dose was adjusted according to the patients’ actual condition. All patients underwent one course of chemotherapy followed by a repeat bone marrow aspiration to assess the efficacy. Due to the difference in treatment plan and prognosis between M3 and other types of AML, patients with M3 subtype were excluded in this study.

Complete remission (CR) was calculated after the first induction therapy, and patients were considered to be in CR if they met the following criteria: (i) no clinical manifestations of anemia, hemorrhage, infection, and leukemic cells infiltration; (ii) hemoglobin ≥100 g/L (male) or 90 g/L (female), absolute neutrophil value ≥1.5 × 10^9^/L, platelets ≥100 × 10^9^/L, and no leukemic cells in peripheral blood classification; (iii) bone marrow blasts plus early stage cells (or juvenile cells) <5%, normal red blood cells, and giant cells [32]. No remission (NR) was defined as failure to meet the above criteria in bone marrow, hemogram, and clinical index after treatment.

### 2.3 Statistical analysis

SPSS software version 25.0 and GraphPad Prism^TM^ 8.02 were employed to analyze the data. While continuous variables were described using medians and ranges, categorical variables were summarized using frequency counts and percentages. The duration from the patient’s diagnosis to the last follow-up or death endpoint was called overall survival (OS). The Log-rank test was employed to assess group differences, and the Kaplan–Meier method was utilized to examine survival data. The data were analyzed using univariate and multivariate Cox proportional hazard regression models. The multivariate analysis to evaluate OS included variables with *p* < 0.05 in the univariate analysis. Statistical significance was determined by a two-sided *p* value < 0.05.


**Ethical approval:** This research was approved by the Affiliated Hospital of Jiangnan University’s Ethics Committee.

## 3 Results

### 3.1 Mutation rate, type, and general characteristics of the *PTPN11* ^mut^ AML

In a cohort of 232 adult AML patients, mutations in the *PTPN11* gene were found in 7.76% (18/232). The median age of *PTPN11* ^mut^ and *PTPN11* ^wt^ patients were 46.5 years (19–66) and 48 years (18–72), respectively, with no significant difference (*p* = 0.7). ten male patients and eight female patients with *PTPN11* mutation, 115 male and 99 female patients with wild type (*p* = 0.882). The white blood cell counts of *PTPN11* ^mut^ and *PTPN11* ^wt^ patients at the first diagnosis were 37.08 (2.58–156.1) × 10^9^/L and 12.36 (0.5–350.75) × 10^9^/L, respectively, with no significant difference (*p* = 0.094). However, hemoglobin and platelet counts of *PTPN11* ^mut^ patients were significantly higher than those of *PTPN11* ^wt^ patients (97.5(62–149) g/L vs 88.5(33–142) g/L, *p* = 0.032), (98(14–713) × 10^9^/L vs 35.5(4–478) × 10^9^/L, *p* < 0.001). No significant difference in bone marrow blasts was observed between the two groups (63.8 (30–95)% vs 55 (6–99.5)%, *p* = 0.052) (Table 1).

**Table 1:** Eighteen *PTPN11* gene mutation and clinical index in AML patients

Case no./sex/age (year)	WBC (×10^9^/L)	HB (g/L)	PLT (×10^9^/L)	FAB	Karyotype	*PTPN1* ^mut^ a.a.change
1/Male/48	11.9	94	713	M5	46, XY	G503A
2/Female/53	63.46	76	33	M5	45, XX, −7,9q-	E76G
3/Female/19	156.1	90	303	M5	46, XX	A72P
4/Male/21	76.24	110	92	M4	46, XY	T73I
5/Female/22	25.5	80	51	M2	46, XX	D61N
6/Female/27	2.58	96	69	M5	46, XX	E76Q
7/Male/32	3.08	111	149	M5	46, XY	T59A
8/Male/33	34.16	62	59	M5	45, XY, Inv(3) (q21q26), −7	D61V
9/Male/38	9.4	109	272	M5	46, XY, t (9;21) (q21; q22)	S502L
10/Male/38	89.19	101	14	M1	46, XY, Del(9) (q13;q22)	A72T
11/Female/45	62.77	95	332	M4	46, XX	A72T
12/Female/55	2.67	116	102	M2	48, XX, +8, Inv(16) (p13;q22)	V45L
13/Male/56	14.12	149	112	M5	47, XY, +8, Inv(9) (p11q22)	F285S
14/Male/56	86.0	106	119	M5	46, XY	G503A
15/Male/56	41.23	146	85	M0	46, XY, t(6,11)	E76K
16/Female/63	64.67	77	33	M5	44, XX, t(2,8) (q35;q13), –21	N58Y, E76K, E76G
17/Female/65	40.0	99	94	M5	47, XX, +21	G60R
18/Male/66	5.9	84	190	M5	46, XY	Q79R

WBC, white blood cell count; HB, hemoglobin; PLT, platelet; FAB, French–American–British classification systems.

**Figure 1:**
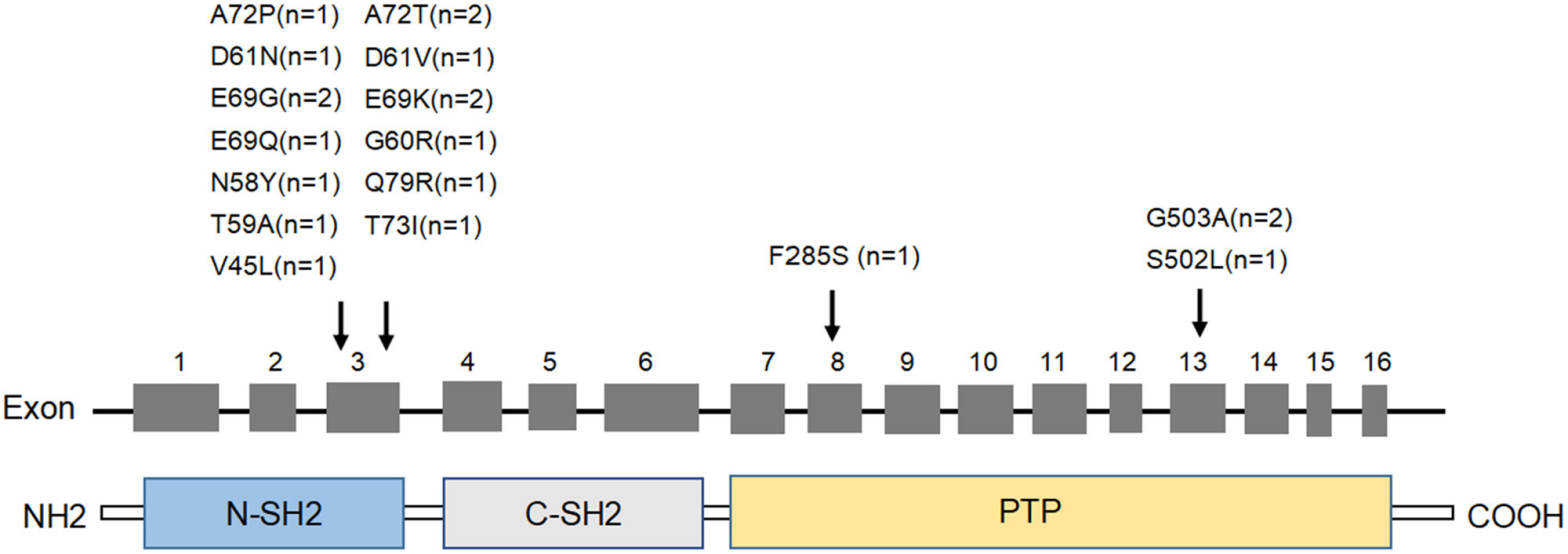
Schematic of *PTPN11* mutation location found in AML patients.

In 18 *PTPN11* ^mut^ patients, there were 20 mutation sites detected in exons 3, 8, and 13, and all of which were missense mutations. These included exon 3 (*n* = 16, 1 with A72P, 2 with A72T, 1 with D61N, 1 with D61V, 2 with E69G, 2 with E69K, 1 with E69Q, 1 with G60R, 1 with N58Y, 1 with Q79R, 1 with T59A, 1 with T73I, 1 with V45L), exon 8 (*n* = 1, 1 with F285S), and exon 13 (*n* = 3, 2 with G503A, 1 with S502L).The N-SH2 and PTP structural domains, respectively, had 16 and 4 mutant sites, and were mainly concentrated in exon 3. No.16 patient had three mutant sites, N58Y, E76K, and E76G mutations in exon 3 (Figure 1).

### 3.2 FAB subtypes of *PTPN11* ^mut^AML

Among all patients, the rate of *PTPN11* mutations was notably higher in M2, M4, and M5 subtypes than in other subtypes (Table 2). In particular, *PTPN11* mutations occurred more frequently in patients with M5 subtype (*p* < 0.001). Out of the 18 *PTPN11* ^mut^ patients, 11.1% (*n* = 2) and 66.67% (*n* = 12) were found in M2 and M5, respectively, whereas 5.56% (*n* = 3) and 5.56% (*n* = 1) were observed in M0 and M1, no *PTPN11* mutations were found in the remaining M6 and M7 subtypes. The status of the remaining 11 patients was unknown.

**Table 2:** Clinical features of *PTPN11* ^mut^ and *PTPN11* ^wt^

Variable	Total (*n* = 232)	*PTPN11* ^mut^ (*n* = 18)	*PTPN11* ^wt^ (*n* = 214)	*p*
**Sex**				
Male, *n*(%)	125(53.9%)	10(10/18, 55.6%)	115(53.7%)	0.882
Female, *n*(%)	107(46.1%)	8(8/18, 44.4%)	99(46.3%)	
**Age (year)**				
Median (range)	48(18–72)	46.5(19–66)	48(18–72)	0.7
**WBC (×10** ^**9**^ **/L)**				
Median (range)	13.8(0.5–350.75)	37.08(2.58–156.1)	12.36(0.5–350.75)	0.094
**Hb (g/L)**				
Median (range)	90(33,149)	97.5(62–149)	88.5(33–142)	**0.032**
**PLT (×10** ^**9**^ **/L)**				
Median (range)	38(4–713)	98(14–713)	35.5(4–478)	**<0.001**
**BM blasts (%)**				
Median (range)	55.75(6–99.5)	63.8(30–95)	55(6–99.5)	0.052
**Cytogenetic karyotype**				
Normal, *n*(n/N, %)	134	9	125	0.489
Abnormal, *n*(n/N, %)	88	9	79	0.273
NA	10	0	10	
**FAB subtype**				
M0	3(1.29%)	1(5.56%)	2(0.93%)	0.096
M1	23(9.91%)	1(5.56%)	22(10.28%)	0.52
M2	83(35.78%)	2(11.11%)	81(37.85%)	**0.022**
M4	50(21.55%)	2(11.11%)	48(22.43%)	0.263
M5	59(27.57%)	12(66.67%)	47(21.96%)	**<0.001**
M6	3(1.29%)	0	3(1.40%)	0.614
Undetermined	11(4.74%)	0	11(5.14%)	0.325
**Fusion gene**				
Negative	169(72.84%)	14(77.78%)	155(72.43%)	0.626
Positive	53(22.84%)	4(22.22%)	49(22.90%)	0.948
*AML1-ETO*	20(8.62%)	0	20(9.35%)	0.176
*MLL-AF6*	6(2.59%)	2(11.11%)	4(1.87%)	**0.018**
*MLL-AF9*	2(0.86%)	0	2(0.93%)	0.681
*MLL-AF10*	2(0.86%)	1(5.56%)	1(0.47%)	0.374
*CBFβ-MYH11*	13(5.6%)	1(5.56%)	12(5.61%)	0.933
Other	10(43.1%)	0	10(4.67%)	0.350
NA	10(43.1%)	0	10(4.47%)	
**Risk stratification (ELN 2017)**				
Favorable	75(32.33%)	4(22.22%)	71(33.18%)	0.341
Intermediate	89(38.36%)	10(55.56%)	79(36.92%)	0.119
Adverse	68(29.31%)	4(22.22%)	64(29.91%)	0.492

WBC, white blood cell count; HB, hemoglobin; PLT, platelet; BM blasts, bone marrow blasts; FAB, French–American–British classification systems; NA, not available.

### 3.3 Chromosomal karyotype in *PTPN11* ^mut^AML

**Figure 2:**
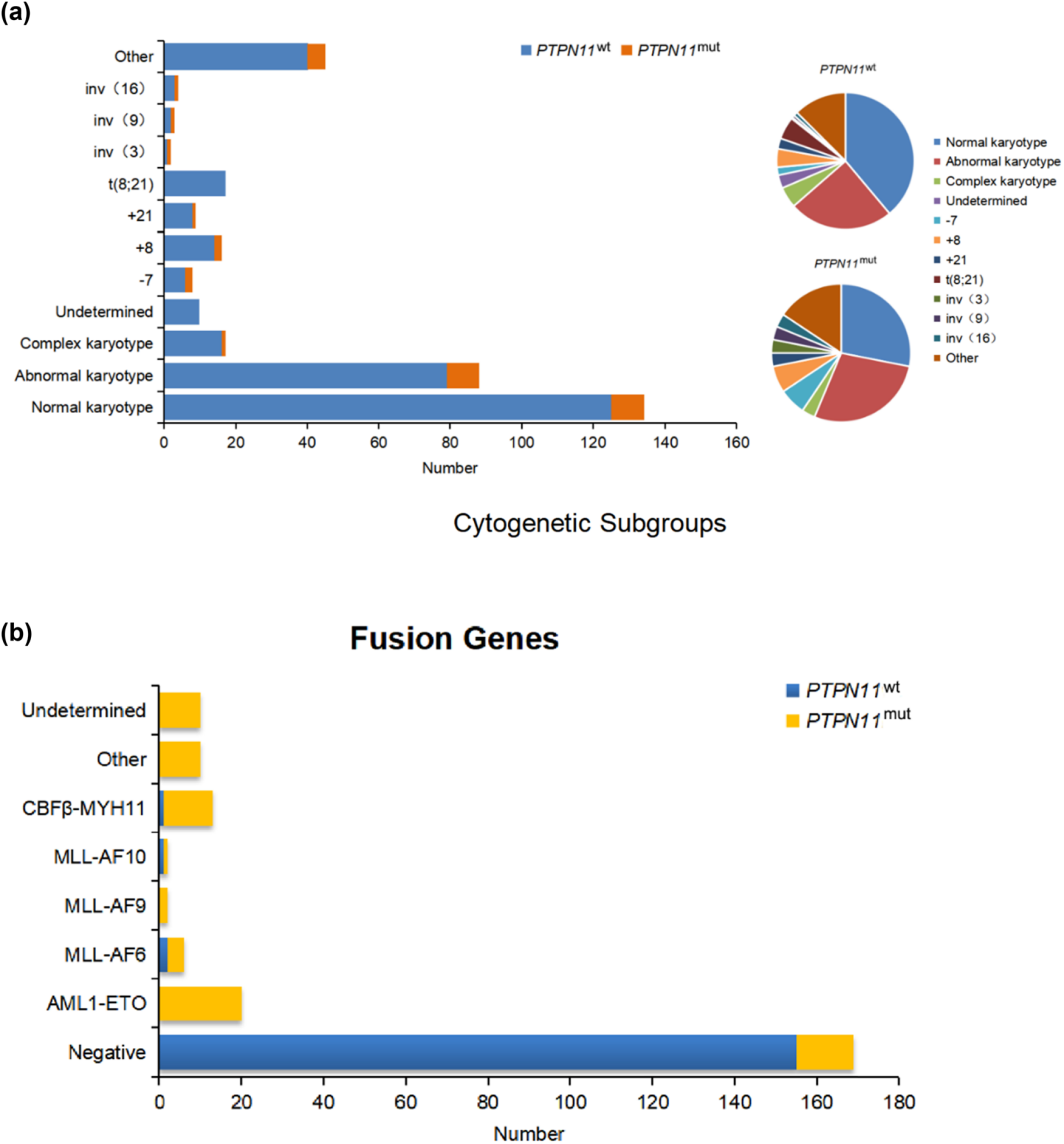
(a) Comparison of the karyotype subgroups of AML patients with *PTPN11* ^mut^ and *PTPN11* ^wt^ and (b) comparison of fusion genes the of AML patients with *PTPN11* ^mut^ and *PTPN11* ^wt^.

Among the 222 cases tested for karyotype, 134 had normal karyotypes and 88 had abnormal karyotypes, including one case of complex karyotype. The incidence of *PTPN11* mutations in normal karyotypes was 6.72% (9/134), and in abnormal karyotypes was 10.23% (9/88). Cytogenetic risk stratification revealed no statistically significant difference in the distribution of *PTPN11* ^mut^ among the favorable-risk (*n* = 4), intermediate-risk (*n* = 10), and adverse-risk (*n* = 4) groups (*p* > 0.05) (Table 2, Figure 2(a)).

### 3.4 Fusion genes in *PTPN11* ^mut^ AML

In this study, a total of 222 cases were examined for available fusion genes, of which 76.13% (169/222) tested negative and 23.87% (53/222) tested positive. All 18 *PTPN11* ^mut^ patients underwent validated fusion gene testing, of which 77.78% (14/18) tested negative and 22.22% (4/18) tested positive. Specifically, *MLL-AF6*, *MLL-AF10*, and *CBFβ-MYH11* accounted for 11.11% (2/18), 5.56% (1/18), and 5.56% (1/18), respectively. Of the 204 *PTPN11* ^wt^ patients, 155 tested negative and 49 tested positive, yielding a positive rate of 24.02% (49/204). The difference between the two groups was not statistically significant (*p* > 0.05). However, AML patients with *MLL-AF6* positive had a higher incidence of *PTPN11* mutation (*p* = 0.018) (Table 2, Figure 2(b)).

### 3.5 Mutations in the *PTPN11* and co-occurring genes

**Figure 3:**
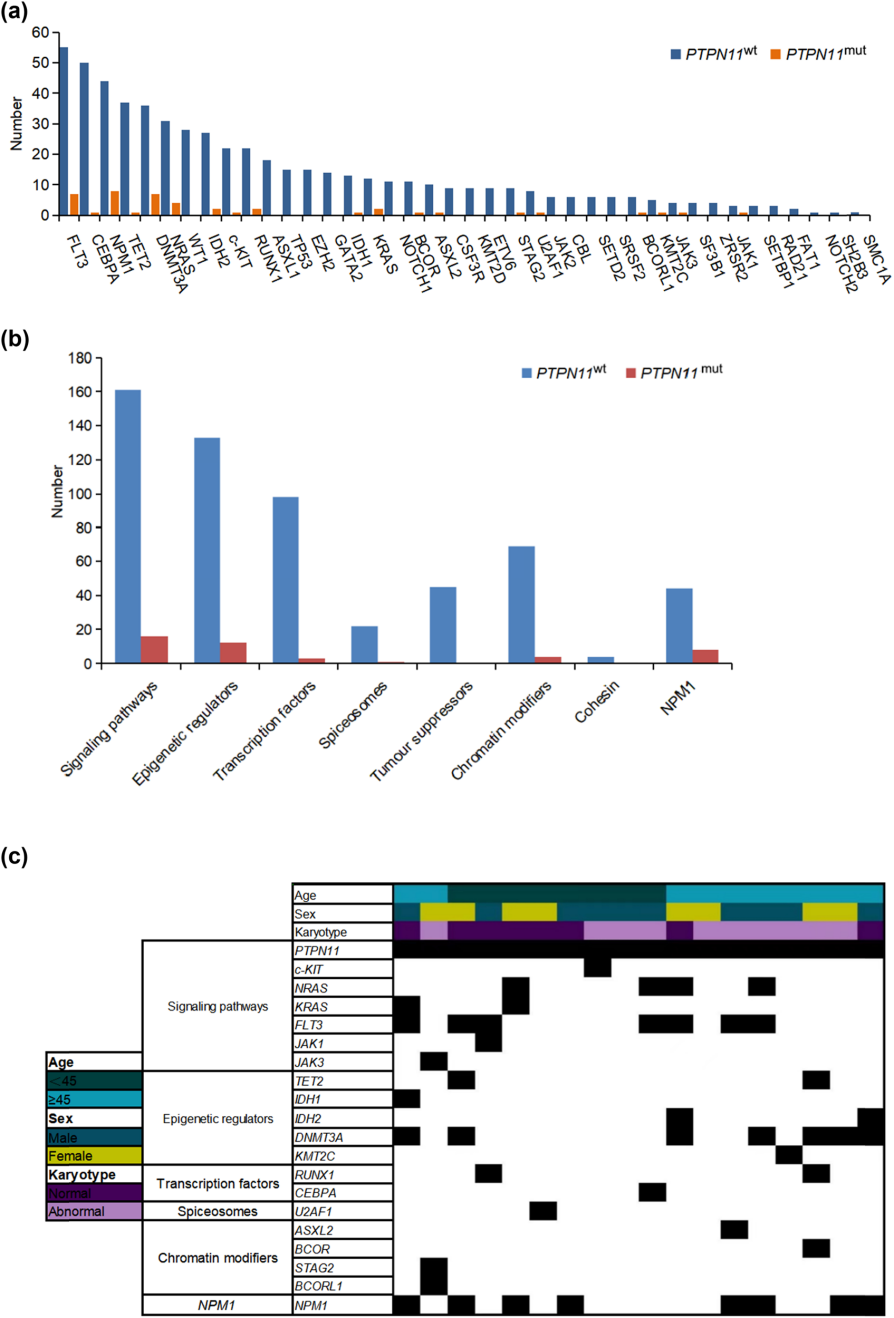
Distribution and functional classification of co-existing mutation genes: (a) comparison of the number of mutations with *PTPN11* ^mut^ and *PTPN11* ^wt^, (b) comparison of the number of mutations in functional genes with *PTPN11* ^mut^ and *PTPN11* ^wt^, and (c) *PTPN11* mutation with co-existing mutation genes in 18 patients (each small grid represents a patient).

In our study, we analyzed the oncogene mutations in adult AML patients (Figure 3(a)), focusing on the *PTPN11* ^wt^ and *PTPN11* ^mut^ groups. Among *PTPN11* ^wt^ patients, the top three genes were *FLT3* (23.7%, 55/232), *CEBPA* (21.55%, 50/232), and *NPM1* (18.97%, 44/232), followed by *TET2* (15.95%, 37/232), *DNMT3A* (15.52%, 36/232), *NRAS* (13.36%, 31/232), *WT1* (12.07%, 28/232), and *IDH2* (11.64%, 27/232). In the *PTPN11* ^mut^ group, the most frequent co-mutated genes were *NPM1*, *DNMT3A*, *FLT3*, *NRAS*, *RUNX1*, *IDH2*, *TET2*, *KRAS*, and *BCORL1*. Interestingly, 88.89% of the *PTPN11* ^mut^ patients had co-existing mutations (Figure 3(c)), with *NPM1* (44.44%, 8/18), *DNMT3A* (38.89%, 7/18), and *FLT3* (38.89%, 7/18) being the most frequent. Five *PTPN11* ^mut^ patients were simultaneously mutated in *NPM1* and *DNMT3A*; however, no *PTPN11* mutations were found to be co-mutated with *KMT2D* and *CSF3R* genes (Table S1).

In addition, 18 patients with *PTPN11* mutations were identified, of which two had a single mutation, four had a double mutation, one had a triple mutation, five had a quadra mutation, and six had greater or equal to penta mutations. The frequency of gene mutation was 3.56 times. We functionally classified the genes in all patients and found that the most common co-mutations were those involved in the RAS Signaling Pathway (88.89%, 16/18), followed by Epigenetic Regulators (66.67%, 12/18), Transcription Factors (16.67%, 3/18), Spliceosomes (5.56%, 1/18), and no Oncogenes or Adhesion protein-related genes. *NPM1* was a separate category with eight cases (44.44%, 8/18). In terms of *PTPN11* ^wt^ patients, the most distributed functional genes were RAS Signaling Pathway related genes (75.23%, 161/214), followed by Epigenetic Regulators (62.15%, 133/214), Transcription Factors (45.79%, 98/214), Chromatin Modifiers (32.24%, 69/214), Oncogenes (21.03%, 45/214), *NPM1* (20.56%, 44/214), Spliceosomes (10.28%, 22/214), and Adhesion Proteins (1.87%, 4/214) (Figure 3(b)).

### 3.6 Response to first induction therapy

We conducted a study to investigate the CR in 217 AML patients, of which 151 achieved CR and 10 cases had *PTPN11* mutations. Among 66 patients who failed to achieve CR, 8 had *PTPN11* mutations. The CR rate between patients with and without *PTPN11* mutation did not show a statistically significant difference (55.56% vs 65.89%, *p* = 0.378). We further divided the patients into two age groups: <60 years old and ≥60 years old. In the <60 years old group (*n* = 194), 132 achieved CR, including 12 with *PTPN11* mutation, while 6% (3/50) of the *PTPN11* mutation patients failed to achieve CR. The CR rate between *PTPN11* ^mut^ and *PTPN11* ^wt^ with no CR showed no statistically significant difference (80% vs 67.04%, *p* = 0.395). In the ≥60 years old group (*n* = 38), 22 achieved CR, including one with *PTPN11* mutation, and 13 failed to achieve CR, two of which were *PTPN11* mutation. Similarly, the CR rate between *PTPN11* ^mut^ and *PTPN11* ^wt^ showed no statistically significant difference (33.33% vs 60%, *p* = 0.562) (Table 3).

**Table 3:** Univariable outcome analyses according to *PTPN11* mutation status

Entire cohort clinical endpoint	Total (*n* = 232)	*PTPN11* ^mut^ (*n* = 18)	*PTPN11* ^wt^ (*n* = 214)	*p*
CR, *n*(%)	151(65.09%)	10(55.56%)	141(65.89%)	0.378
NR, *n*(%)	66(28.45%)	8(44.44%)	58(27.1%)	0.118
NA, *n*(%)	15(6.47%)	0	15(7.01%)	
**OS**				
Median, mo (95% CI)	43(30.6–35.29)	20.0(18.65–36.57)	43(30.95–35.84)	0.2
1-year OS (%)	187(80.6)	14(77.78)	173(80.84)	
3-year OS (%)	123(53.01)	7(38.89)	116(54.21)	
4.5-year OS (%)	9(3.88)	1(5.56)	8(3.74)	
Younger adults Clinical endpoint (18 years ≤ age < 60 years)	Total (*n* = 194)	*PTPN11* ^mut^ (*n* = 15)	*PTPN11* ^wt^ (*n* = 179)	*p*
CR, *n*(%)	132(68.04)	12(80)	120(67.04)	0.395
NR, *n*(%)	50(25.77)	3(20)	47(26.26)	0.764
NA, *n*(%)	12(6.19)	0	12(6.7)	
**OS**				
Median, mo (95% CI)	44(31.8–36.82)	20(19.95–39.92)	44(32.08–37.29)	0.319
1-year OS (%)	162(83.51)	12(80)	150(83.8)	
3-year OS (%)	110(56.7)	7(46.67)	103(57.54)	
4.5-year OS (%)	9(4.64)	1(6.67)	8(4.47)	
Older adults Clinical endpoint (age ≥ 60 years)	Total (*n* = 38)	*PTPN11* ^mut^ (*n* = 3)	*PTPN11* ^wt^ (*n* = 35)	*p*
CR, *n*(%)	22(57.89)	1(33.33)	21(60)	0.562
NR, *n*(%)	13(34.21)	2(66.67)	11(31.43)	0.265
NA, *n*(%)	3(7.89)	0	3(8.57)	
**OS**				
Median, mo (95% CI)	22(19.63–32.21)	15(22.56–54.56)	26(20.12–33.42)	0.401
1-year OS (%)	25(65.79)	2(66.67)	23(65.71)	
3-year OS (%)	13(34.21)	0	13(37.14)	
4.5-year OS (%)	0	0	0	
M5 adults clinical endpoint	Total (*n* = 61)	*PTPN11* ^mut^ (*n* = 12)	*PTPN11* ^wt^ (*n* = 49)	*p*
CR, *n*(%)	49(80.33)	8(66.67)	41(83.67)	**<0.001**
NR, *n*(%)	10(16.39)	4(33.33)	6(12.24)	0.096
NA, *n*(%)	2(3.28)	0	2(4.08)	
**OS**				
Median, mo (95% CI)	32(26.92–36.07)	19.5(14.05–38.12)	33(27.78–37.85)	0.305
1-year OS (%)	47(77.05)	9(75)	38(77.55)	
3-year OS (%)	28(45.9)	4(33.33)	24(48.98)	
4.5-year OS (%)	1(1.64)	1(8.33)	0	

CR, first induction therapy complete remission; NR, no remission; NA, not available; mo, months; OS, overall survival; *p*, *p*-value.

### 3.7 Survival analysis

**Figure 4:**
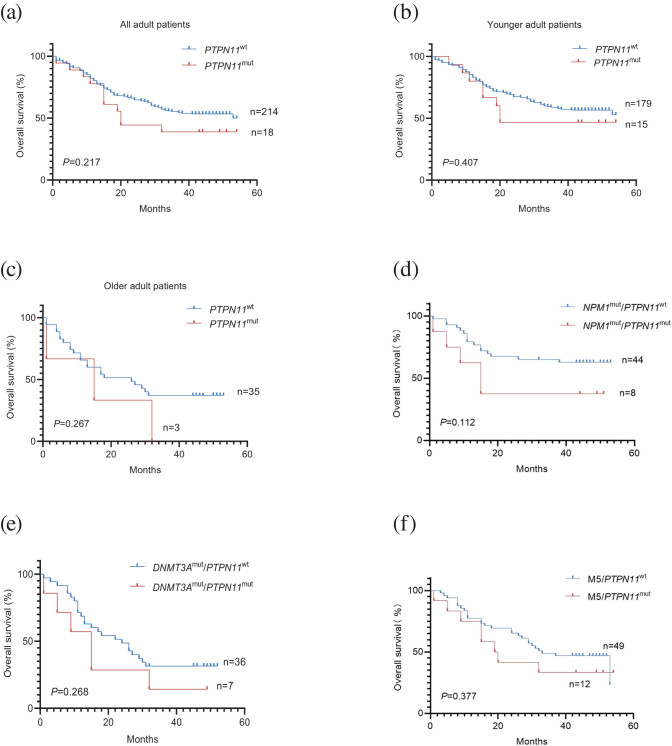
Influence of mutations in *PTPN11* on survival: (a) *Kaplan–Meier* estimates of OS, (b) younger adult patients, (c) older adult patients, (d) *NPM1* ^mut^/*PTPN11* ^mut^, *NPM1* ^mut^/*PTPN11* ^wt^, (e) *DNMT3A* ^mut^/*PTPN11* ^mut^, *DNMT3A* ^mut^/*PTPN11* ^wt^, and (f) M5/*PTPN11* ^mut^, M5/*PTPN11* ^wt^ with adult *de novo* AML.

The median OS was 43 (95% CI: 30.6–35.29) months in all patients. There was no statistically significant difference between *PTPN11* ^mut^ and *PTPN11* ^wt^ patients (20 months, 95% CI: 18.65–36.57 vs 43 months, 95% CI: 30.95–35.84, *p* > 0.05) (Figure 4(a)). We analyzed the OS for the different groups of patients at 1, 3, and 4.5 years. Among all AML patients, 1-year OS was found to be 80.6% (187/232) and 6.03% (14/232) were *PTPN11* ^mut^ patients. Three-year OS was 53.01%, of which 3.03% (7/232) of patients were with *PTPN11* mutations. 3.88% (9/232) of AML patients surviving for 4.5 years, *PTPN11* ^mut^ patients were only one. The median OS in 194 younger AML patients (18 years ≤ age < 60 years) was 44 months (95% CI: 31.8–36.82). Compared with *PTPN11* ^mut^ and *PTPN11* ^wt^ patients, the difference of OS was not significant (20 months, 95% CI: 19.95–39.92 vs 44 months, 95% CI: 32.08–37.29, *p* > 0.05) (Figure 4(b)). 83.51% (162/194) of AML patients had 1-year OS, *PTPN11* ^mut^ patients were 6.19% (12/194). 56.7% (110/232) of patients with 3-year OS, and 3.61% (7/194) *PTPN11* ^mut^ patients achieved 3-year OS. 4.64% (9/194) of AML patients obtained 4.5-year OS, while only one patient with *PTPN11* mutation, less than *PTPN11* ^wt^ patients (*n* = 8). 38 cases of older AML patients (age ≥ 60 years), the OS was 22 months (95% CI: 19.63–32.21). There was no significant difference between *PTPN11* ^mut^ and *PTPN11* ^wt^ patients (15 months, 95% CI: 22.56–54.56 vs 26 months, 95% CI: 20.12–33.42, *p* > 0.05) (Figure 4(c)). The 1-year OS and 3-year OS of the older group were, respectively, 65.79% (25/38) and 34.21% (13/38), while no AML patients with 4.5-year OS were identified. There was one case that achieved 1-year OS with *PTPN11* ^mut^ significantly less than *PTPN11* ^wt^ patients (*n* = 23). No *PTPN11* ^mut^ patients were found with 3-year OS and 4.5-year OS (Table 3).

We also performed a comparative analysis of OS between *NPM1* ^mut^/*PTPN11* ^mut^ and *NPM1* ^mut^/*PTPN11* ^wt^, and finally showed that the median OS was not statistically different between the two groups (15 months vs 45 months, *p* = 0.112) (Figure 4(d)). *DNMT3A* ^mut^/*PTPN11* ^mut^ and *DNMT3A* ^mut^/*PTPN11* ^wt^ were also analyzed, the median OS was found to be with no statistically significant difference (15 months vs 45 months, *p* = 0.268) (Figure 4(e)).

In a univariate analysis of adult *de novo PTPN11* mutation patients, platelet count ≥100 × 10^9^/L, bone marrow blasts ratio ≥65.4%, and co-existence with *DNMT3A* or *IDH2* mutations were found to influence survival (*p* < 0.05) (Table 4).

**Table 4:** Univariate analysis of the OS analysis in adult AML patients with *PTPN11* mutation

Variables	*χ* ^2^	*p*
Sex (male vs female)	0	0.989
Age (18–60 vs ≥60 years)	2.301	0.129
WBC (<50 vs ≥50 × 10^9^/L)	0	0.983
HB (<110 vs ≥110 g/L)	0.477	0.490
PLT (<100 vs ≥100 × 10^9^/L)	1.716	**0.019**
BM blasts (<65.4 vs ≥65.4%)	4.938	**0.026**
Cytogenetic karyotype (normal vs abnormal)	3.805	0.051
FAB subtype (*n* [%])	0.482	0.975
M0	0.099	0.753
M1	0.027	0.87
M2	0.12	0.729
M4	1.498	0.221
M5	0.017	0.895
Fusion gene	0	0.992
*MLL-AF6* (positive vs negative)	0.511	0.475
*MLL-AF10* (positive vs negative)	0.099	0.753
*CBFβ-MYH11* (positive vs negative)	2.503	0.114
Risk stratification (adverse vs favorable/intermediate)	1.798	0.18
CR (yes vs no)	0.187	0.665
*NPM1* (mutated vs wild type)	0.645	0.422
*DNMT3A* (mutated vs wild type)	4.126	**0.042**
*FLT3* (mutated vs wild type)	0.124	0.725
*NRAS* (mutated vs wild type)	0.373	0.541
*KRAS* (mutated vs wild type)	0.124	0.725
*RUNX1* (mutated vs wild type)	0.288	0.591
*IDH2* (mutated vs wild type)	4.985	**0.026**
*TET2* (mutated vs wild type)	0.122	0.727

WBC, white blood cell; HB, hemoglobin; PLT, platelet; CR, first induction therapy complete remission; *p*, *p*-value.

Multivariate Cox proportional hazard regression model was conducted with survival as the outcome variable (no = 0, yes = 1), incorporating significant factors from the univariate analysis as predictors. These factors included platelet count (<100 × 10^9^/L = 0, ≥100 × 10^9^/L = 1), bone marrow blast percentage (<65.4% = 0, ≥65.4% = 1), *DNMT3A* mutation status (wild type = 0, mutated = 1), and *IDH2* mutation status (wild type = 0, mutated = 1). The result revealed that bone marrow blasts percentage ≥65.4% was regarded as an independent predictor of prognosis (*p* < 0.05) (Table 5).

**Table 5:** Multivariate analysis for OS in *PTPN11* mutation

Variables	HR (95% CI)	*p*
PLT (<100 vs ≥100 × 10^9^/L)	0.555 (0.211–1.458)	0.232
BM blasts (<65.4 vs ≥65.4%)	3.192 (1.038–9.814)	**0.043**
*DNMT3A* (mutated vs wild type)	0.443 (0.144–1.369)	0.157
*IDH2* (mutated vs wild type)	0.334 (0.057–1.950)	0.223

OS, overall survival; HR, hazard ratio; CI, confidence interval; *p*, *p*-value.

### 3.8 Outcome of *PTPN11* mutation with M5 subtypes

In our study, we found that the FAB typing of patients with *PTPN11* mutation mostly showed M5 subtype (*p* < 0.05), so 61 patients with M5 subtype were further analyzed. Among them, 19.67% (*n* = 12, 12/61) had *PTPN11* ^mut^ patients and 80.33% (*n* = 49, 49/61) had *PTPN11* ^wt^ patients. The CR rate was lower in *PTPN11* ^mut^ patients than in wild-type patients (66.67%, 8/12 vs 83.67%, 41/49, *p* < 0.001). Regarding OS with the M5 subtype patients, there was no statistically significant difference between *PTPN11* ^mut^ patients and *PTPN11* ^wt^ patients (19.5 months, 95% CI: 14.05–38.12 vs 33 months 95% CI: 27.78–37.85, *p* > 0.05). 77.05% (47/61) patients had 1-year OS, with 75% (9/12) of *PTPN11* ^mut^ patients and 77.55% (38/49) of wild-type patients. Among all M5 subtypes, 3-year OS was 33.33% (4/12) and 48.98% (24/49) for *PTPN11* mutant patients and wild-type patients, respectively. 8.33% (1/12) *PTPN11* ^mut^ patients had 4.5-year OS while *PTPN11* ^wt^ patients had none (Table 4, Figure 4(f)).

## 4 Discussion

This study revealed a mutation rate of 7.76% in the *PTPN11* gene among AML patients (none APL). The majority of these mutations were observed in the M5 subtype according to FAB classification. Additionally, the *PTPN11* mutations often co-existed with *NPM1* and *DNMT3A* mutations. However, no significant impact on prognosis was observed as a result of these mutations. In M5 subtype AML patients, *PTPN11* mutations do not affect the OS of the patients. Besides, a bone marrow blast percentage ≥65.4% was identified as an independent factor influencing the OS of patients with *PTPN11* mutations. The innovation of this study lies in the grouping of AML patients into *PTPN11* ^mut^ and *PTPN11* ^wt^ groups, allowing for a comparison of the clinical characteristics between these two groups in adult AML (none APL) patients. In addition, the analysis of the co-occurrence of *PTPN11* mutations with *NPM1* and *DNMT3A* mutations, as well as the impact of the M5 subtype, on the prognosis of adult AML patients was performed. This research has clinical significance as it contributes to a better understanding of adult AML, improves prognostic assessment, and enhances OS outcomes for patients.

The *PTPN11* gene encodes the Non-Receptor Tyrosine Kinase Protein SHP2 which is involved in key signaling functions in normal hematopoiesis, such as proliferation, differentiation, and apoptosis [7]. The molecular structure of SHP2 consists of two SH2 domains at the N-terminal and the PTP activity region at the C-terminal [33]. Studies have shown that mutations in the N-SH2/PTP domain can lead to leukemic transformation by up-regulating SHP2 activity and inducing hypersensitivity to GM-CSF, as well as over-activating the RAS signaling axis [34].

This study aimed to explore the clinical characteristics of adult *PTPN11* gene mutation and its impact on the prognosis of adult AML patients. It has been found that *PTPN11* mutations are not limited to adult AML, but are also associated with a range of hematological malignancies, such as JMML, childhood AML, myelodysplastic syndromes, and acute B-lymphocytic leukemia [21,35,36]. In addition, *PTPN11* mutations have also been linked to the occurrence of solid tumors (e.g., carcinoma of the lungs, hepatic cell carcinoma, breast, ovarian, gastric, and prostate cancers) [12] and NS [37]. Furthermore, the *PTPN11* gene has been identified as a drug target for the intrinsic and acquired drug resistance of cancer drugs, which has important implications for clinical treatment [38].

The incidence of *PTPN11* mutations in adult AML patients in our study was 7.76%, comparable to the results from previous studies [26,39]. Of the 18 patients with *PTPN11* mutations, 20 different mutation sites were identified, all of which were missense mutations. The majority (16/20) of mutation sites were located in the N-SH2 structural domain encoded by exon 3, with A72T, E69G, and E69K being the most frequent. G503 was found to be more frequent in exon 13, which is consistent with other research on AML [25,40]. The frequency of mutations in exons 8 and 13 was lower than that of N-SH2, at 20.6% (22/107) and 17.8% (19/107), respectively [37]. This suggested that the *PTPN11* mutation in adult AML may be variable in terms of mutational sites.

In our study, *PTPN11* ^mut^ and *PTPN11* ^wt^ patients were compared in terms of white blood cell, hemoglobin, and platelet counts. The results showed that there was no statistically significant difference in white blood cell counts between the two groups (37.08 × 10^9^/L vs 12.36 × 10^9^/L, *p* > 0.05). However, *PTPN11* ^mut^ patients had significantly higher hemoglobin levels than *PTPN11* ^wt^ patients (97.5 g/L vs 88.5 g/L, *p* = 0.032). Additionally, platelet counts were significantly higher in *PTPN11* ^mut^ patients than in *PTPN11* ^wt^ patients (35.5 × 10^9^/L vs 98 × 10^9^/L, *p* < 0.001), which was in agreement with the former studies [26,41].There was no statistically significant difference in terms of gender, age, and bone marrow blasts between the two groups (*p* > 0.05). Moreover, the *PTPN11* mutation was mainly detected in the M5 subtype of childhood AML [21], which is consistent with previous findings. These findings indicate that the *PTPN11* mutation might be related to monocyte differentiation.

Our study showed that the median age of adult AML patients with *PTPN11* ^mut^ and *PTPN11* ^wt^ did not differ significantly (46.5(19–66) years vs 48(18–72) years, *p* > 0.05), which was consistent with previous research [26,41]. Moreover, the *PTPN11* mutation in this study occurred mostly in the normal karyotype, and notably, one case of complex karyotype was found in *PTPN11* mutation with an incidence of 5.56% [26]. This finding was in line with previous studies that identified one *PTPN11* mutation associated with inv(3) (q21q26), which was associated with a marker of poor prognosis in AML patients concerning future malignant transformation [26,42].

Fusion genes have been identified as specific molecular markers of acute leukemia, and *PTPN11* mutations have been observed to co-exist with *MLL-AML* (*MLL-AF6*, *MLL-AF10*) and *CBFβ-MYH11*, which is in accordance with previously reported findings in the literature [43,44]. Furthermore, evidence suggests that the RAS pathway is hyper-activated in childhood AML patients with *MLL-AF6* positive, and that *PTPN11*, as an important regulator of the RAS pathway, may contribute to this activation by causing SHP2 to remain active in the RAS pathway [45]. Thus, a connection between *PTPN11* mutation and *MLL-AF6* is conceivable, although further confirmation is necessary.

Our study has revealed that *PTPN11* mutations are often associated with other genes mutations with a rate lower than 5%, including *TP53*, *CSF3R*, and *GATA2*, which is consistent with the findings of Stasik et al. [39]. Similarly, Alfayez et al. [41] also identified that *PTPN11* mutations are susceptible to co-exist with *NPM1* and *DNMT3A* mutations. *NPM1* is a protein ubiquitously expressed in the nucleolus and holds favorable prognostic significance [24]. This study demonstrated that, although PTPN11 mutations frequently co-occurred with *NPM1* mutations, they did not have an impact on prognosis, which is consistent with the findings of Liu et al. [25]. Patients with *DNMT3A* gene mutations in AML have been associated with poorer prognosis. Furthermore, *DNMT3A* is another gene that is prone to co-occur with *PTPN11* mutations [27,28]. To investigate this further, we analyzed the OS of AML patients with *DNMT3A* ^mut^/*PTPN11* ^mut^ and *DNMT3A* ^mut^/*PTPN11* ^wt^, and found that *DNMT3A* gene mutation did not have an impact on the prognosis of *PTPN11* ^mut^ patients (*p* ＞ 0.05).

Similar to the findings of Swoboda et al., our investigation showed that there was no discernible difference in the CR rate between patients with *PTPN11* ^mut^ and *PTPN11* ^wt^ [46]. However, the prognostic value of *PTPN11* mutations in AML remains controversial. Alfayez et al. [41] reported that *PTPN11* ^mut^ patients had lower overall remission rates (ORR) and CR rates than *PTPN11* ^wt^ patients in *de novo* AML (67% vs 82%, *p* = 0.03; 44% vs 71%, *p* = 0.006), while no influence on ORR and CR was identified between *PTPN11* ^mut^ and *PTPN11* ^wt^ patients in refractory relapsing AML. Notably, our results showed that *PTPN11* mutations were mainly located in the M5 subtype (*p* < 0.05). To further investigate the clinical significance of the M5 subtype in *PTPN11* ^mut^ patients, we separately evaluated the CR and OS of AML patients with the M5 subtype. Consequently, we found that the CR rate of *PTPN11* ^mut^ patients was lower than *PTPN11* ^wt^ patients (66.67% vs 82.67%, *p* < 0.001). This indicates that the response to the first induction therapy in *PTPN11* ^mut^ patients is associated with the M5 subtype. Moreover, the M5 subtype did not affect the OS of *PTPN11* ^mut^ patients (*p* > 0.05). To the best of our knowledge, this is the first investigation into how *PTPN11* mutations in the M5 subtype affect clinical outcomes.

Contrary to earlier research, there was no significant difference in median OS between *PTPN11* ^mut^ and *PTPN11* ^wt^ in this study (20 months, 95% CI: 19.95–39.92 vs 44 months, 95% CI: 32.08–37.29, *p* > 0.05) [39]. This discrepancy may be due to the limited sample size, resulting in an undetectable outcome. Additionally, we separated all the patients into two age groups: 18 ≤ *y* < 60 years and *y* ≥ 60 years. This finding contrasted those of Fobare et al. [26] and revealed that there was no statistically significant difference in median OS between *PTPN11* ^mut^ and *PTPN11* ^wt^ in the 18–60-year-old group (20 months, 95% CI: 19.95–39.92 vs 44 months, 95% CI: 32.08–37.29, *p* > 0.05). In the ≥60 years group, the difference was still not significant between *PTPN11* ^mut^ and *PTPN11* ^wt^ (15 months, 95% CI: 22.56–54.56 vs 26 months, 95% CI: 20.12–33.42, *p* > 0.05), which was in accordance with the results of Fobare et al. [26].

We sought to identify factors affecting OS in patients with *PTPN11* ^mut^ associated AML. By analyzing relevant clinical features and concomitant gene mutations, we identified the bone marrow blasts ratio ≥65.4% as an independent prognostic factor, which can change how normally functioning hematopoietic stem cells behave and play a significant part in the genesis of AML [47]. Accordingly, consideration of the bone marrow blasts ratio is paramount in the clinical management of *PTPN11* ^mut^ associated AML patients.

## 5 Conclusions

*PTPN11* mutations were found to occur in 7.76% of adult *de novo* AML patients (none APL), all of which were missense mutations, with exon 3 mutations being the most frequent. Patients carrying *PTPN11* mutations were more likely to be of the M5 subtype, and had higher hemoglobin and platelet levels, as well as a lower CR rate compared to *PTPN11* ^wt^ patients. Furthermore, the frequency of *PTPN11* mutations was higher in patients with *MLL-AF6* positive AML. In addition, *PTPN11* mutations were most often present in conjunction with *NPM1* and *DNMT3A* mutations, though these had no prognostic impact. It was discovered that an independent factor affecting OS for *PTPN11* ^mut^ individuals was the percentage of bone marrow blasts ratio 65.4%. In order to develop a theoretical framework for future clinical prognostic classification and treatment, future research should increase the sample size to further investigate the clinical significance and gene function of *PTPN11* mutations.

## Supplementary Material

Supplementary Table
