# Supplementary material for: Analysis of the clinical characteristics and prognosis of adult de novo acute myeloid leukemia (none APL) with PTPN11 mutations
Source: Open Med (Wars). 2023 Nov 3;18(1):20230830. doi: 10.1515/med-2023-0830 (PMC10655689; doi:10.1515/med-2023-0830)
Supplement: Supplementary Table [file med-2023-0830-sm.pdf]

Supplementary material

Table S1: Mutation analysis of *PTPN11* mutations and wild-type AML patients

Mutational genes	Total (*n* = 232)	*PTPN11*^{mut} (*n* = 18)	*PTPN11*^{wt} (*n* = 214)	*P*
**Signalings pathway, n (%)**	177(76.29%)	16(88.89%)	161(75.23%)	0.875
c-KIT	23(9.91%)	1(5.56%)	22(10.28%)	0.520
NRAS	35(15.09)	4(22.2%)	31(14.49%)	0.379
KRAS	14(6.03%)	2(11.11%)	12(5.61%)	0.347
FLT3	62(26.72%)	7(38.89%)	55(25.7%)	0.226
CSF3R	9(3.88%)	0	9(4.21%)	0.376
NOTCH1	11(4.74%)	0	11(5.14%)	0.326
NOTCH2CH2	1(0.43%)	0	1(0.47%)	0.772
JAK1	4(1.72)	1(5.56%)	3(1.4%)	0.194
JAK2	6(2.59%)	0	6(2.8)	0.473
JAK3	5(2.16%)	1(5.56%)	4(1.87%)	0.302
SH2B3	1(0.43%)	0	1(0.47%)	0.772
CBL	6(2.59%)	0	6(2.8%)	0.473
**Epigenetic regulators, n (%)**	149(64.22%)	16(88.89%)	133(62.15%)	0.761
TET2	39(16.81%)	2(11.11%)	37(17.29%)	0.502
IDH1	14(6.03%)	1(5.56%)	13(6.07%)	0.929
IDH2	29(12.5%)	2(11.11%)	27(12.62%)	0.853
DNMT3A	43(18.53%)	7(38.89%)	36(16.82%)	0.021
KMT2C	6(2.59%)	1(5.56%)	5(2.33%)	0.41
KMT2D	9(3.88%)	0	9(4.21%)	0.376
SETD2	9(3.88%)	3(16.67%)	6(2.8%)	0.004
**Spiceosomes, n (%)**	23(9.9%)	1(5.56)	22(10.28%)	0.520
SRSF2	6(2.59%)	0	6(2.8%)	0.473
SF3B1	4(1.72%)	0	4(1.87%)	0.559
U2AF1	9(3.88%)	1(5.56%)	8(3.74%)	0.702
ZRSR2	4(1.72%)	0	4(1.87%)	0.559
**Transcription factors, n (%)**	101(43.53%)	3(16.67%)	98(45.79%)	0.035
RUNX1	24(10.34%)	2(11.11%)	22(10.28%)	0.912
ETV6	9(3.88%)	0	9(4.21%)	0.376
GATA2	14(6.03%)	0	14(6.54%)	0.264

(Continued)

Table S1: *Continued*

**Mutational genes**	**Total (*n* = 232)**	***PTPN11*^{mut} (*n* = 18)**	***PTPN11*^{wt} (*n* = 214)**	***P***
SETBP1	3(1.29)	0	3(1.40%)	0.614
CEBPA	51(21.98%)	1(5.56%)	50(23.36%)	0.08
**Tumour suppressors, n (%)**	45(19.4%)	0	45(21.03%)	0.041
TP53	15(6.47%)	0	15(7.01%)	0.246
WT1	28(12.07%)	0	28(13.08%)	0.103
FAT1	2(0.86%)	0	2(0.93%)	0.681
**Chromatin modifiers, n (%)**	73(31.47%)	4(22.22%)	69(32.24%)	0.316
ASXL1	18(7.76%)	0	18(8.41%)	0.201
ASXL2	11(4.74%)	1(5.56%)	10(4.67%)	0.866
EZH2	15(6.47%)	0	15(7.0%)	0.246
BCOR	12(5.17%)	1(5.56%)	11(5.14%)	0.939
STAG2	10(4.31%)	1(5.56%)	9(4.21%)	0.787
BCORL1	7(3.02%)	1(5.56%)	6(2.8%)	0.513
**Cohesin, n (%)**	4(1.72%)	1(5.56%)	4(1.87%)	0.559
RAD21	3(1.29%)	0	3(1.4%)	0.614
SMC1A	1(0.43%)	1(5.56%)	0	0.772
***NPM1*, n (%)**	52(22.41%)	8(44.4%)	44(20.56%)	0.02
